# Complete Genome Sequence of *Geobacter* sp. Strain FeAm09, a Moderately Acidophilic Soil Bacterium

**DOI:** 10.1128/MRA.00979-20

**Published:** 2021-01-14

**Authors:** Pooja Yadav, Sanjay Antony-Babu, Erin Hayes, Olivia M. Healy, Donald Pan, Wendy H. Yang, Whendee L. Silver, Christopher L. Anderson, Adam Voshall, Samodha C. Fernando, Etsuko N. Moriyama, Joshua R. Herr, Karrie A. Weber

**Affiliations:** a School of Biological Sciences, University of Nebraska—Lincoln, Lincoln, Nebraska, USA; b Department of Plant Biology, University of Illinois at Urbana-Champaign, Urbana, Illinois, USA; c Department of Geology, University of Illinois at Urbana-Champaign, Urbana, Illinois, USA; d Department of Environmental Science, Policy, and Management, University of California, Berkeley, Berkeley, California, USA; e Department of Animal Science, University of Nebraska—Lincoln, Lincoln, Nebraska, USA; f Center for Plant Science Innovation, University of Nebraska—Lincoln, Lincoln, Nebraska, USA; g Department of Plant Pathology, University of Nebraska—Lincoln, Lincoln, Nebraska, USA; h Department of Earth and Atmospheric Sciences, University of Nebraska—Lincoln, Lincoln, Nebraska, USA; i Daugherty Water for Food Institute, University of Nebraska—Lincoln, Nebraska, USA; Queens College

## Abstract

A moderately acidophilic *Geobacter* sp. strain, FeAm09, was isolated from forest soil. The complete genome sequence is 4,099,068 bp with an average GC content of 61.1%. No plasmids were detected. The genome contains a total of 3,843 genes and 3,608 protein-coding genes, including genes supporting iron and nitrogen biogeochemical cycling.

## Abstract (precis)

A moderately acidophilic *Geobacter* sp. strain, strain FeAm09, was isolated from forest soil. The complete genome sequence is 4,099,068 bp, with an average GC content of 61.1%. No plasmids were detected. The genome contains a total of 3,843 genes and 3,608 protein-coding genes, including genes supporting iron and nitrogen biogeochemical cycling.

## ANNOUNCEMENT

Microbial reduction of iron [Fe(III)] oxide minerals plays a significant role in coupled biogeochemical cycles in unsaturated soils (1, 2) and in saturated soils and sediments (3–6). To date, very few Fe(III)-reducing bacteria have been isolated from unsaturated soils. Here, we report the complete genome of *Geobacter* sp. strain FeAm09, which was isolated as an Fe(III)-reducing bacterium from moderately acidic unsaturated tropical forest soil in Puerto Rico (7) following enrichment in piperazine-*N*,*N*′-bis(3-propanesulfonic acid) (PIPPS)-buffered medium (pH 5.0) amended with synthetic ferrihydrite (8) under an anoxic atmosphere, transfer to medium containing Fe(III)-nitrilotriacetic acid (NTA), and isolation with anaerobic shake tubes (80:20 N_2_/CO_2_ atmosphere) (9, 10).

**FIG 1:**
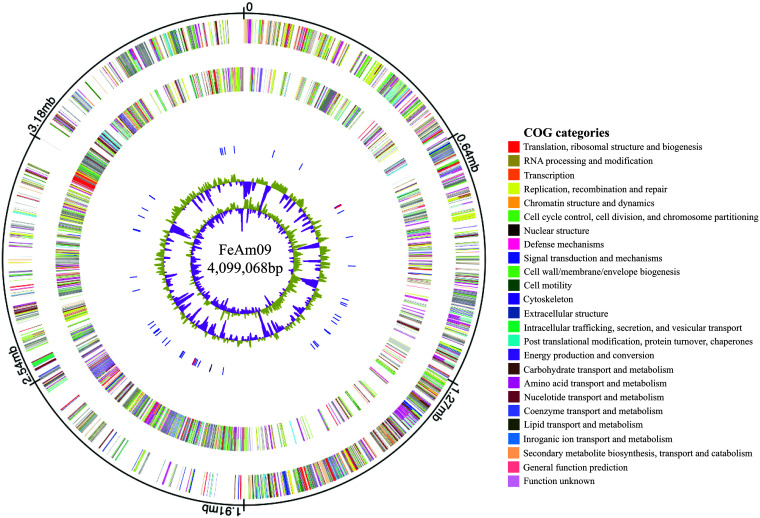
Circular genome map of *Geobacter* sp. strain FeAm09, generated by using DNAPlotter from Artemis version 18.1.0 (Wellcome Sanger Institute) (21). From the outside to the center: circle 1, DNA base position; circle 2, genes on the forward strand (color by Clusters of Orthologous Groups of proteins [COG] category); circle 3, genes on the reverse strand (color by COG category); circle 4, RNA genes (tRNAs, blue; rRNAs, red; other RNAs, black); circle 5, GC content plotted using a 10-kb window size (purple represents values below average, while olive represents values above average); circle 6, GC skew [(G + C)/(G – C)] plotted using a 10-kb window size (purple indicates values below average, while olive represents values above average).

Cells of *Geobacter* sp. strain FeAm09 were cultivated with fumarate (40 mM) and acetate (20 mM) under an anoxic atmosphere (100% argon). Cultures were incubated at 37°C in the dark until mid-log phase (approximately 48 h), harvested by centrifugation (5 min at 12,000 × *g*), and resuspended in DNA extraction buffer (11). Cells were immediately lysed by heating (95°C for 15 min). Genomic DNA from the lysate was extracted using phenol-chloroform-isoamyl alcohol (25:24:1) followed by ethanol precipitation (12). PacBio 20-kb SMRTbell library preparation (P6-C4 chemistry) using the PacBio RS platform was completed by the University of Delaware DNA Sequencing and Genotyping Center. A total of 45,767 reads with a mean read length of 12,898 nucleotides were assembled into a single contig using the PacBio Hierarchal Genome Assembly Process (HGAP) pipeline version 3.0 with default parameters in single-molecule real-time (SMRT) Portal version 2.3.0 (13). The assembly revealed a single circular chromosome with a GC content of 61.1%. Genome annotation using PGAP version 4.8 predicted 3,843 total genes (14, 15). Totals of 6 rRNAs, 52 tRNAs, 1 transfer-messenger RNA, and 3,608 protein-coding sequences were identified (Fig. 1).

*Geobacter* sp. strain FeAm09 belongs to the *Geobacteraceae* family and is a Gram-negative bacterium. Similar to other known Fe(III)-reducing *Geobacter* spp., the annotated genome of strain FeAm09 revealed putative genes for *c*-type cytochromes and type IV pili. The only nitrate reduction genes identified in the genome were for dissimilatory nitrate reduction to ammonium (nitrate reductase and nitrite reductase). Genes annotated for reduction of dimethyl sulfoxide (DMSO), thiosulfate, and sulfite were also identified, as were cytochrome *bd* ubiquinol terminal oxidase (*cydB* and *cydA*), similar to Geobacter sulfurreducens (16, 17). Hemerythrin and genes associated with oxidative stress tolerance (catalase peroxidase and rubrerythrin) were also identified. In addition to genes that support heterotrophy through the use of organic electron donors (formate dehydrogenase and citrate lyase), a NiFe hydrogenase (*hydB*) capable of converting H_2_ to H^+^ was found, which would enable lithotrophic growth. Genes associated with the reverse tricarboxylic acid (TCA) cycle (*mdh*, *fumB*, *frdA*, *frdB*, *sucC*, *sucD*, *korA*, *korB*, *icdI*, *acnB*, and *citC*) were identified, which could support autotrophy (18–20). Genes linked to assimilatory sulfate reduction and nitrogen fixation were also identified. Together, these physiological capabilities of *Geobacter* sp. strain FeAm09 indicate a diverse lifestyle that can adapt to fluctuating environmental conditions commonly found in soil systems.

### Data availability.

The genome sequence was deposited in GenBank under BioProject PRJNA555606, with the accession numbers CP042466 and SRX9278424.
